# Heterogeneity of PD-L1 Expression Between the Primary Tumor and Matched Lymph Node Metastases in Head and Neck Squamous Cell Carcinomas

**DOI:** 10.3390/cancers18081286

**Published:** 2026-04-18

**Authors:** Moritz Knebel, Gilbert Georg Klamminger, Jan Philipp Kühn, Sandrina Körner, Silke Wemmert, Lukas Alexander Brust, Felix Braun, Sigrun Smola, Mathias Wagner, Martin Ertz, Luc G. T. Morris, Bernhard Schick, Maximilian Linxweiler

**Affiliations:** 1 Institute of Otorhinolaryngology, Saarland University, 66421 Homburg, Germany; jan.kuehn@uks.eu (J.P.K.); sandrina.koerner@uks.eu (S.K.); silke.wemmert@uks.eu (S.W.); lukas.brust@uks.eu (L.A.B.); felix.braun@uks.eu (F.B.); bernhard.schick@uks.eu (B.S.); maximilian.linxweiler@uks.eu (M.L.); 2 Department of Otorhinolaryngology, Head and Neck Surgery, Saarland University Medical Center, 66421 Homburg, Germany; 3 Department of General and Surgical Pathology, Saarland University Medical Center, 66421 Homburg, Germany; gilbert.klamminger@unimedizin-mainz.de (G.G.K.); mathias.wagner@uks.eu (M.W.); martin.ertz@uks.eu (M.E.); 4 Department of Obstetrics and Gynecology, University Medical Center of the Johannes Gutenberg University Mainz, 55131 Mainz, Germany; 5 Institute of Virology, Saarland University Medical Center, 66421 Homburg, Germany; sigrun.smola@uks.eu; 6 Helmholtz Institute for Pharmaceutical Research Saarland (HIPS), Helmholtz Centre for Infection Research, 66123 Saarbrücken, Germany; 7 Department of Surgery, Memorial Sloan Kettering Cancer Center, New York City, NY 10065, USA; morrisl@mskcc.org; 8 Laboratory of Experimental Cancer Immunogenomics, Memorial Sloan Kettering Cancer Center, New York City, NY 10065, USA

**Keywords:** HNSCC, PD-L1 expression, spatial heterogeneity, lymph node metastases, immunotherapy, tumor microenvironment

## Abstract

**Background**: The role of immune checkpoint inhibition in treating head and neck squamous cell carcinoma (HNSCC) is expanding, yet response rates to PD-L1 therapy remain inconsistent and generally poor. Although several studies have examined heterogeneous intratumoral PD-L1 expression, the disparity in response to PD-L1 therapy between primary tumors and their associated lymph node metastases remains unclear. **Methods**: Primary tumor samples and two matching lymph node metastases were obtained from a cohort of 50 patients and immunohistochemically stained with a PD-L1 antibody. PD-L1 expression, assessed using the combined positive score (CPS) and tumor proportion score (TPS), and immune infiltration, measured with an immunoreactive score (IRS), were compared between the primary tumor and lymph node metastases. These measures were then correlated with other histopathological and clinical features. **Results**: PD-L1 expression, evaluated by CPS and TPS, showed no significant differences between the primary tumor and matched lymph node metastases. Discordance relative to established regulatory cut-offs was observed in a subset of patients, affecting 18% (CPS; 95% CI, 8.0–30.0%) and 4% (TPS; 95% CI, 0.0–10.0%) of cases. CPS and TPS values were not influenced by primary tumor subsite or HPV status. Conversely, immune infiltration measured by IRS was significantly affected by primary tumor subsite location. Both HPV tumor status and primary tumor subsite were statistically significantly associated with overall survival. **Conclusions**: Our findings highlight variability in PD-L1 expression in HNSCC and may offer context for differential responses of primary tumors and lymph node metastases to immune checkpoint therapy reported in recent clinical studies. These observations support the need for a more comprehensive characterization of PD-L1 expression across tumor sites in head and neck cancer. Further investigation is required to determine whether, and in which settings, reassessment of PD-L1 status in metastatic lesions—including lymph node metastases—may provide additional clinically relevant information when initial testing does not meet established therapeutic cut-offs.

## Simple Summary

Immunotherapy has emerged as an important treatment modality for patients with head and neck squamous cell carcinoma; however, only a minority achieve durable clinical benefit, and discordant response patterns between primary tumors and lymph node metastases have been reported. Because the use of anti-PD-1 therapy remains contingent on PD-L1 expression according to regulatory approval criteria, we investigated whether spatial heterogeneity in PD-L1 expression exists between primary tumors and matched lymph node metastases and whether such differences might contribute to variable treatment responses. Our analysis indicated that 18% of patients (CPS; 95% CI, 8.0–30.0%) exhibited discordance in PD-L1 expression between primary and metastatic sites when assessed relative to current therapeutic cut-offs. These findings highlight the potential for spatial heterogeneity in PD-L1 expression and underscore an area of uncertainty in biomarker classification. As such, they should be interpreted as hypothesis-generating. Future studies may be warranted to determine whether, and under what circumstances, reassessment of PD-L1 status in metastatic lesions—including lymph node metastases—could provide additional clinically relevant information, particularly in cases where initial testing does not meet established eligibility thresholds for anti-PD-1 therapy.

## 1. Introduction

Head and neck squamous cell carcinoma (HNSCC) is the seventh most common type of cancer worldwide, with 890,000 estimated new cases annually [1]. Major risk factors include chronic alcohol and tobacco consumption as well as infection of the oral/pharyngeal mucosa with high-risk human papillomavirus (HPV) [2]. Over decades, the prognosis of HNSCC patients has not significantly improved, with the five-year overall survival rate persisting at 50–60% [3]. Even if initial treatment is successful, which in most cases encompasses a multimodal approach including surgery, radiation, and/or radio-chemotherapy, more than 50% of patients develop recurrent or metastatic disease within five years after first diagnosis [4].

In this context, the introduction of immune checkpoint inhibition into the therapeutic management of various human cancer entities, such as HNSCCs, marked a milestone in clinical oncology and revolutionized our understanding of cancer treatment [5,6]. The introduction of the anti-PD-1 antibodies Pembrolizumab and Nivolumab raised hopes for better overall survival in patients with recurrent or metastatic tumors, but response rates to immune checkpoint therapy remain poor, with only 20–30% of patients experiencing long-term benefit [7]. Regarding the clinical approval of the aforementioned antibodies by federal agencies, prognostic indicators such as a tumor proportion score (TPS) > 50% and a combined positive score (CPS) ≥ 1 have been identified for predicting a good immunotherapeutic response [8]. In the United States, Pembrolizumab monotherapy is approved for the first-line treatment of recurrent or metastatic HNSCC in patients whose tumors express programmed death ligand 1 (PD-L1) with a CPS ≥ 1. Additionally, a combination of Pembrolizumab with platinum and 5-fluorouracil (5-FU) chemotherapy can be chosen for treatment regardless of PD-L1 expression [9]. Similarly, tumor mutational burden (TMB) was included as a companion biomarker in the United States’ Food and Drug Administration (FDA) approval, where patients with TMB ≥ 10 mutations/megabase can qualify for Pembrolizumab treatment [10]. In contrast, approval by the European Medicines Agency (EMA) is restricted to tumors with CPS ≥ 1 for both Pembrolizumab monotherapy and its combination with chemotherapy or a TPS > 50% in second-line treatment [6]. In addition to varying authorization policies and therefore different prognostic biomarkers, numerous studies have examined heterogeneous intratumoral PD-L1 expression to account for the poor and inconsistent response rates [11,12]. With respect to treatment response, only a few studies to date have investigated the differential response rates between primary tumors and lymph node metastases. The IMCISION trial, a non-randomized phase Ib/IIa study involving 32 HNSCC patients treated with two doses of immune checkpoint blockade (ICB) using Nivolumab or Nivolumab plus a single dose of Ipilimumab prior to surgery, demonstrated a highly discordant response between the primary tumor and lymph node metastases as measured by PET-CT activity following immunotherapy [13].

Because PD-L1 expression level in both tumor cells and tumor-associated immune cells is a clinically relevant biomarker guiding the use of anti-PD-1 antibodies within the current standard of care, and because clinical studies have documented discordant therapeutic responses between primary tumors and corresponding lymph node metastases, we investigated PD-L1 expression in paired primary tumors and matched lymph node metastases from a cohort of 50 patients with head and neck squamous cell carcinoma (HNSCC) treated at our institution. This analysis was undertaken to determine whether spatial heterogeneity in PD-L1 expression within individual patients could influence therapeutic decision-making in the recurrent or metastatic setting. Therefore, this study sought to assess whether discordant patterns of PD-L1 expression between primary and metastatic sites might contribute to the variability in response rates to immune checkpoint blockade and to explore the rationale for refining eligibility criteria for immunotherapy.

## 2. Materials and Methods

### 2.1. Patients and Tissue Samples

For the immunohistochemical analyses, primary tumor tissue samples, as well as two matched lymph node metastasis samples, were obtained from 50 HNSCC patients. All patients underwent curative intent surgical treatment. This cohort included 88% male and 12% female patients with a mean age of 62.6 years. Details of the patients’ demographic and clinical data are shown in Table 1. Clinical and TNM stages were defined according to the 8th version of the AJCC/UICC head and neck cancer staging system [14]. All patients were diagnosed and treated at the Saarland University Medical Center (Homburg, Germany). Tumor and lymph node tissue from the patients was obtained during surgical tumor resection and neck dissection surgery. Median patient follow-up was 37.5 months. The Saarland Medical Association ethics review committee approved the scientific use of the patients’ tissue and clinical data (index number 218-10). All experiments were performed according to the relevant guidelines and regulations. Written informed consent was obtained from all patients.

### 2.2. Immunohistochemistry

Immunohistochemistry was utilized to analyze PD-L1 expression, measured by the CPS and TPS, as well as immune infiltration, assessed using an immunoreactive score (IRS). Tumor tissue was formalin-fixed, paraffin-embedded, and sectioned into 4 µm slices with a Leica RM 2235 rotation microtome (Leica Microsystems, Wetzlar, Germany). The slices were transferred to adhesive slides (TOMO, Matsunami, Osaka, Japan) using a 46 °C water bath and dried overnight at 37 °C. Hematoxylin and Eosin (H&E) staining was performed on each tissue sample for morphological control according to standard protocol. For immunohistochemical detection of PD-L1, slides were incubated using the automated Benchmark Ultra system (Ventana Medical Systems, Roche, Basel, Switzerland) and a mouse monoclonal primary antibody specific for PD-L1 (Dako/Agilent, Santa Clara, CA, USA; clone 22C3; dilution 1:25) for 32 min at 37 °C. The bound antibody was visualized using ultraView Universal Alkaline Phosphatase Red Detection (Roche, Basel, Switzerland), following the manufacturer’s instructions. Heat-induced antigen retrieval was performed at 97 °C with CC1 buffer (Ventana, Roche, Basel, Switzerland) for 64 min. Each staining series included negative controls by omitting the primary antibody, and on-slide positive controls (validated human tonsil tissue) were used. Immunohistochemically stained tumors and matching lymph node metastases were evaluated using the ‘Tumor Proportion Score’ (TPS) and the ‘combined positive score’ (CPS). Here, TPS reports the percentage of membranously stained tumor cells in relation to all vital tumor cells, whereas CPS calculates a relation of membranously stained tumor cells plus membranously or cytoplasmatically stained mononuclear immune cells (macrophages, lymphocytes, dendritic cells) to all vital tumor cells. Only tumors with >100 vital cells were considered; non-neoplastic epithelial cells, ganglionic cells, fibroblasts, or areas of necrosis were not taken into account [15]. During staining interpretation, particular attention was devoted to the evaluation of PD-L1 expression within affected lymph nodes, as accurate morphological identification of relevant structures is inherently critical in this context. Tumor cells were distinguished from surrounding immune cells based on established morphological criteria (e.g., cytological features and growth patterns), with H&E staining used for spatial correlation where necessary. In accordance with good clinical practice, constitutive PD-L1 expression in germinal centers and other pre-existing lymph node structures was excluded from analysis. Scoring was performed in line with established criteria for the applied scoring system (CPS/TPS, see description above), ensuring that tumor cell-associated staining was evaluated specifically, while inflammatory cells were only considered when appropriate (namely, within the intratumoral and peritumoral stroma, defined as the connective tissue component within the field of view at 20× magnification). Additionally, stained slides were semiquantitatively assessed using the IRS according to Remmele and Stegner [16]. First, the absolute number of immune cells in the tumor and peritumoral tissue was valued from 1 to 4 (1—no immune cells; 2—<25%; 3—25–50%; 4—>50% of tissue infiltrated with stained immune cells) and the relative number of positively stained immune cells was valued from 1 to 3 (1—<30%; 2—30–60%; 3—>60%). Both values were multiplied, resulting in a score ranging from 1 to 12. Three examiners, including one board-certified pathologist, independently analyzed each IHC staining, remaining blinded to the clinical diagnosis, HPV status, and each other’s scoring.

### 2.3. HPV Tumor Status

The HPV tumor status of the included patients was assessed using a combination of immunohistochemical p16 staining and HPV-DNA-PCR. Among the n = 50 HNSCC patients in our study, 72% tested negative for HPV, while 28% tested positive. Only patients with both positive p16 IHC and positive HPV-DNA-PCR were assigned a positive HPV tumor status. Regarding the significantly worse prognosis and different tumor cell biology of discordant (p16−/HPV+ or p16+/HPV−) HNSCC patients, the proof of a positive p16- and positive HPV-DNA PCR testing was mandatory for assigning positive HPV tumor status [17,18]. For HPV-DNA-PCR, DNA was extracted from fresh-frozen tumor tissue using the QIAamp DNA Blood Mini Kit (Qiagen, Hilden, Germany) following the manufacturer’s instructions. Then, HPV-DNA-PCR was performed with the LightCycler 2.0 (Roche Diagnostics, Mannheim, Germany) using GP5+/6+ primers as described previously [19]. PCR amplification products were detected with SYBR Green as well as gel electrophoresis. Following an initial denaturation at 95 °C for 15 min, 45 PCR cycles were performed with denaturation at 95 °C for 10 s, annealing at 45 °C for 5 s, and elongation at 72 °C for 18 s. After amplification of the PCR products, a melting curve analysis was conducted with temperatures between 45 °C and 95 °C, with a rise in temperature of 0.2 °C/s. Every PCR analysis included a HPV16-positive control (Tm 79 °C) and a HPV18-positive control (Tm 82 °C). Additionally, the Glyceraldehyde-3-phosphat-dehydrogenase (GAPDH) gene was amplified and used as an internal positive control [20]. For immunohistochemical detection of p16, the CINtec p16 histology kit (Roche Diagnostics) was used according to the manufacturer’s instructions. In brief, heat-induced epitope unmasking was performed upon deparaffinization in a rice cooker for 20 min using the supplied retrieval buffer. Incubation with the p16 antibody and the detection of staining signals were performed as recommended by the manufacturer. Every staining series included negative and positive controls.

### 2.4. Statistical Analysis

For statistical analyses, the D’Agostino & Pearson omnibus normality test, Anderson-Darling test, Shapiro–Wilk test, and Kolmogorov–Smirnov test were used to determine if the datasets followed a Gaussian distribution in each comparison. Gaussian distribution was only assigned if the data sample passed ≥2 of the aforementioned normality tests. If the data showed a normal distribution, parametric tests were performed (two-tailed unpaired *t*-tests, one-way ANOVA with Tukey’s correction for multiple comparisons, or Pearson correlation). If the data showed no normal distribution, non-parametric tests were applied (Mann–Whitney-U test, one-way ANOVA using Kruskal–Wallis with Dunn’s correction for multiple comparisons, or Spearman correlation). For repeated measurements, mixed-effects models (REML), fitted for each outcome variable (IRS intratumoral, IRS peritumoral, CPS, TPS), with tissue sample (PT, M1, M2) specified as a fixed effect and subject as a random effect, were conducted. Sphericity was not assumed; the Geisser-Greenhouse correction was applied. Post hoc pairwise comparisons were performed using Tukey’s test. Statistical significance was set at α = 0.05. Interobserver reproducibility of PD-L1 scoring was evaluated using Fleiss’ kappa. CPS was categorized as <1, 1–20, and >20, and TPS was categorized as <25%, 25–50%, 51–75%, and >75%. Agreement was calculated across all 150 specimens assessed independently by three observers. For the analysis of concordance and discordance in PD-L1 staining, bootstrap resampling was performed, and agreement was quantified using Cohen’s kappa statistic. Additionally, multivariable Cox proportional hazards models were fitted as an exploratory analysis to account for confounding factors (age, sex, T stage, UICC stage, HPV status, PD-L1 expression (CPS ≥ 1), and immune infiltration (IRS low)). No formal adjustment for multiple comparisons was applied. For survival analyses, a log-rank test was used. *p* values < 0.05 were considered statistically significant (α = 0.05).

## 3. Results

### 3.1. Correlation of PD-L1 Expression Between the Primary Tumor and Matching Lymph Node Metastases

**Figure 1:**
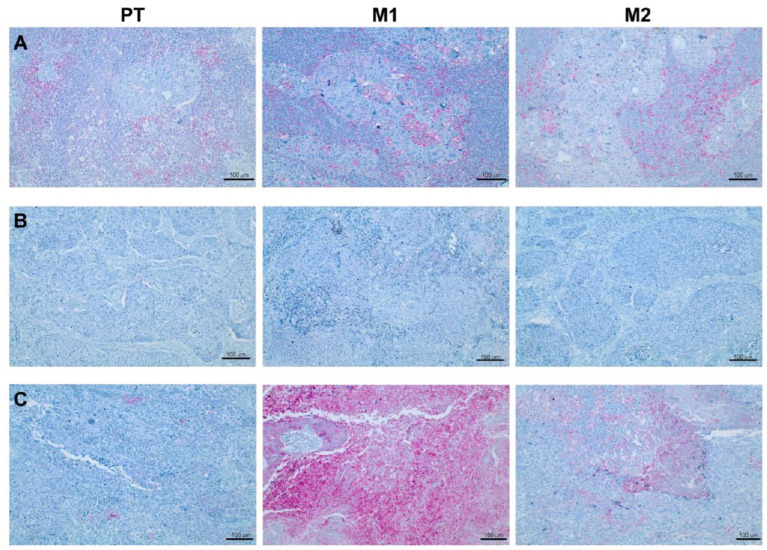
Immunohistochemical analysis of PD-L1 expression. Representative immunohistochemical stainings are shown for (**A**) one concordant case with combined positive score (CPS) values ≥ 1 in the primary tumor as well as in both lymph node metastases, (**B**) one concordant case with CPS values < 1 and (**C**) one discordant case with CPS value < 1 in the primary tumor but CPS values ≥ 1 in both lymph node metastases.

**Figure 2:**
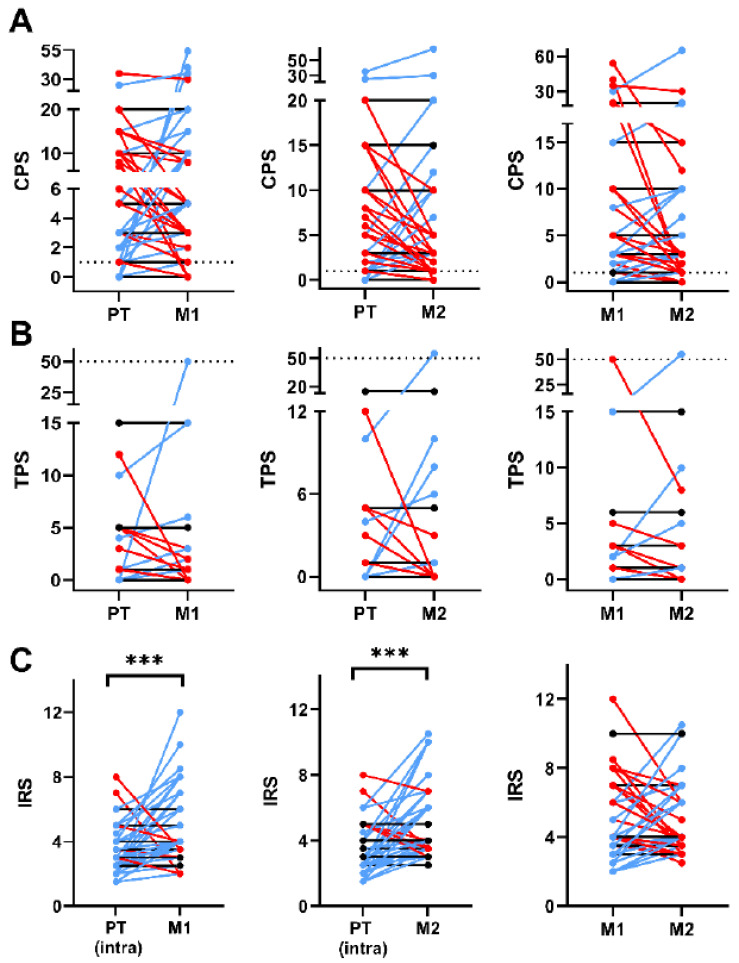
Comparison of combined positive score (CPS) (**A**), tumor proportion score (TPS) (**B**) and immunoreactive score (IRS) (**C**) between the primary tumor and matched lymph node metastases. In (**A**–**C**), blue lines indicate an increase in values while red lines indicate a decrease in values from the primary tumor (PT) to the lymph node metastases or from metastasis one (M1) to metastasis two (M2), respectively. Black lines indicate a stable value. ***: *p* < 0.001.

To evaluate a potentially heterogeneous PD-L1 expression between the primary tumor and lymph node metastases, immunohistochemical staining was performed and evaluated using CPS and TPS, exemplarily shown in Figure 1. Comparisons of CPS (F(1.6, 79.7) = 1.20, *p* = 0.30) and TPS (F(1.9, 93.2) = 0.29, *p* = 0.74) values between the primary tumor and respective metastases showed no significant differences (Figure 2). However, CPS values between primary tumors and metastases, as well as between the metastases themselves, showed a relevant heterogeneous distribution in single cases, with values both above and below CPS = 1 in the same patient (Figure 2A).

However, TPS values > 50%, which could make patients eligible for immunotherapy in a second-line therapeutic setting, were found less frequently. Interobserver agreement was substantial for CPS (κ = 0.78) and TPS (κ = 0.72). In addition to PD-L1 expression, immune cell infiltration was measured using a modified immunoreactive score (IRS) by Remmele and Stegner [16]. A significant increase in IRS values was observed in the lymph node metastases compared to the primary tumor’s intratumoral area (F(2.0, 97.4) = 13.54, *p* < 0.0001; PT vs. M1 *p* = 0.0003; PT vs. M2, *p* < 0.0001; Figure 2C). For example, in one patient, we observed a CPS value of 15 in the primary tumor and CPS values below 1 in both lymph node metastases. Additionally, we noted heterogeneous CPS values in another patient, with a CPS below 1 in the primary tumor, CPS of 54 in the first lymph node metastasis, and CPS of 12 in the second lymph node metastasis. Similar patterns were observed for TPS (primary tumor < 1, metastasis 1 = 50, metastasis 2 = 8) and IRS (primary tumor = 7, metastasis 1 = 2, metastasis 2 = 3.5). Pairwise agreement of PD-L1 classification between sampling sites was assessed using Cohen’s kappa. For CPS, agreement was moderate between the primary tumor and both lymph node metastases (κ = 0.545 for PT vs. LK1 and PT vs. LK2) and substantial between the two metastases (κ = 0.750). In contrast, the kappa values for TPS were close to zero; however, their interpretability is limited by the highly imbalanced distribution, with most TPS values below 50%.

Regarding the prognostic implications of the aforementioned subgroups, our analysis revealed a trend toward reduced patient survival in cases with discordant CPS values. In contrast, both concordant CPS values above and below 1 were associated with improved overall survival. Discordant TPS values exhibited a non-significant trend toward improved overall survival, in contrast to concordant TPS values below 50% (see Supplementary Figure S1).

**Figure 3:**
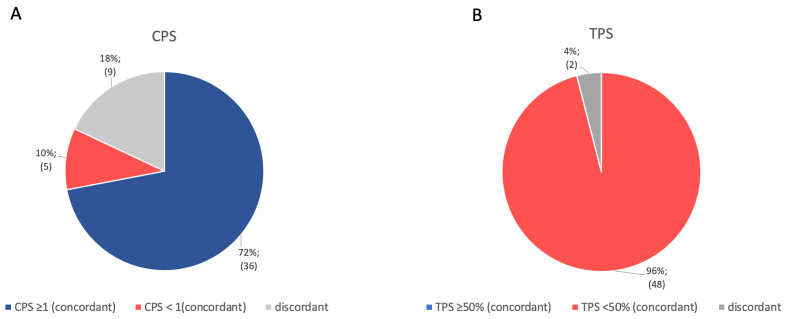
Distribution of the heterogeneous PD-L1 expression of our collective (n = 50) between the primary tumor and matched lymph node metastases, shown by (**A**) combined positive score (CPS) and (**B**) tumor proportion score (TPS). Blue represents concordant cases with CPS values ≥ 1/TPS ≥ 50%, both in the primary tumor and the two lymph node metastases. Red represents concordant cases with CPS values < 1/TPS < 50%, both in the primary tumor and the two lymph node metastases. Gray describes the cases in which values in the primary tumor and the lymph node metastases are discordant (e.g., primary: CPS ≥ 1, M1 CPS < 1, M2 CPS < 1). Both relative proportions and corresponding absolute case numbers are presented alongside each fraction.

Figure 3 highlights the clinical relevance of the observed heterogeneous PD-L1 expression of tumor cells and tumor infiltrating immune cells, revealing that 18% (CPS; 95%-CI: 8.0–30.0%) and 4% (TPS; 95%-CI: 0.0–10.0%) of patients in our cohort had discordant PD-L1 expression, meaning they would only be eligible for immune checkpoint therapy if the sample was taken from a specific site. However, the majority of patients showed concordant levels of PD-L1 expression. Specifically, 72% and 10% of patients had concordant CPS levels below or above 1 in all three samples, respectively. Consistently, 96% of patients had TPS values below 50% in both primary tumor and lymph node metastases samples.

### 3.2. Correlation of PD-L1 Status with Clinical and Histopathological Characteristics

**Figure 4:**
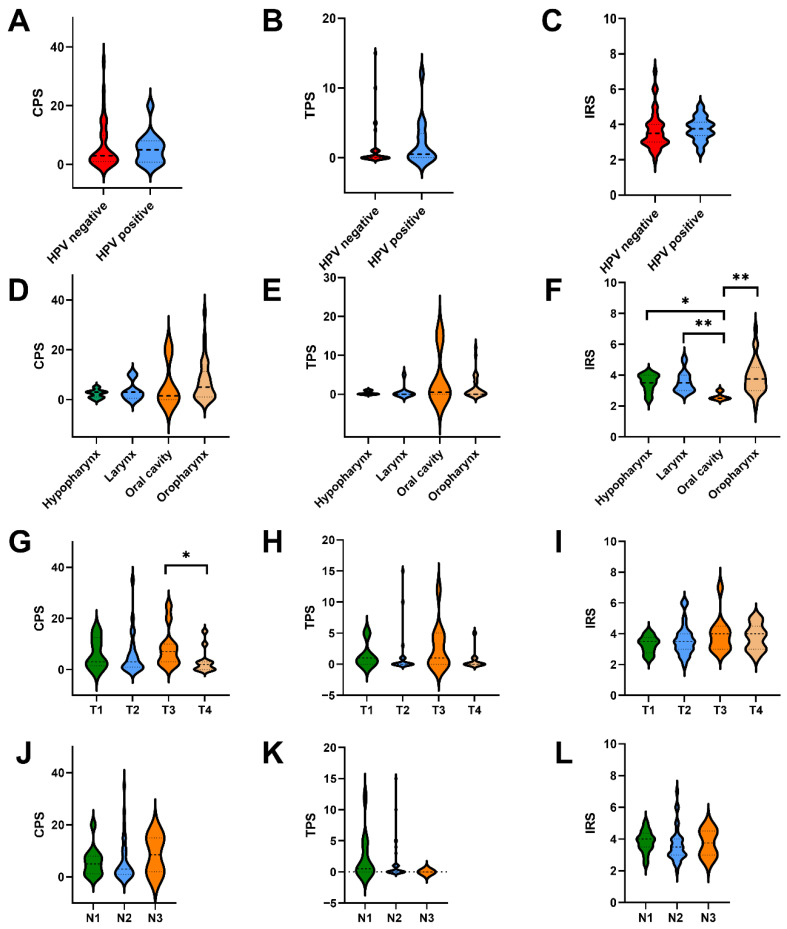
Correlation of combined positive score (CPS), tumor proportion score (TPS), and peritumoral immunoreactive score (IRS) of the primary tumor with clinical and histopathological features such as HPV status (**A**–**C**), primary tumor localization (**D**–**F**), T-stage (**G**–**I**), and N-stage (**J**–**L**). In (**A**–**L**), the median is shown by a horizontal line. * = *p* < 0.05, ** = *p* < 0.01.

Clinical characteristics such as HPV status, TNM stages, and primary tumor localization were correlated with PD-L1 expression (CPS, TPS, IRS). CPS and TPS were not significantly influenced by HPV tumor status (Figure 4A,B), although a slight tendency towards higher CPS values was observed in HPV-positive cases. Additionally, a tendency towards higher infiltration with PD-L1-positive immune cells was observed in HPV-positive cases (Figure 4C). An additional subgroup analysis restricted to oropharyngeal carcinomas did not demonstrate a significant association between HPV tumor status and PD-L1 expression, irrespective of whether expression was assessed using CPS or TPS (Supplementary Figure S2). Primary tumor localization had no significant influence on PD-L1 expression measured by CPS and TPS (Figure 4D,E), but significantly correlated with the PD-L1 IRS, with higher IRS values in the oropharynx, larynx, and hypopharynx as compared to the oral cavity (Figure 4F). Conversely, T- and N-stages showed no significant association with PD-L1 expression, except for CPS levels when comparing T3 with T4 stages (Figure 4G–L).

### 3.3. Impact of PD-L1 Expression on Patients’ Outcome

**Figure 5:**
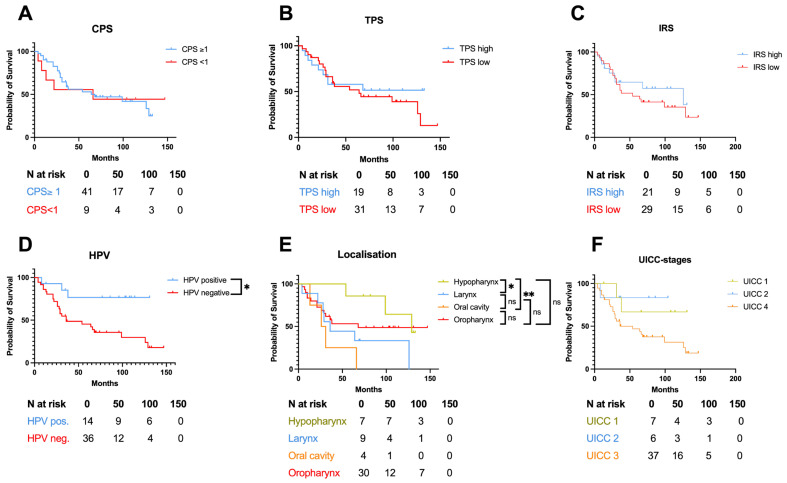
Prognostic relevance of combined positive score (CPS), tumor proportion score (TPS), and peritumoral immunoreactive score (IRS) of the primary tumor, HPV tumor status, primary tumor localization, and UICC-stages. (**A**) Patients’ overall survival regarding CPS values ≥ 1 and <1. (**B**,**C**) Correlation of overall survival with TPS values and peritumoral IRS values regarding high and low values, defined by the median. (**D**) Patients’ overall survival segregated by HPV tumor status. (**E**) Overall survival depending on localization of the respective primary tumor site. (**F**) Patient overall survival regarding different UICC stages. In (**A**–**F**), a log-rank test was used for statistical analysis. The number of patients at risk is provided in the corresponding table. * = *p* < 0.05, ** = *p* < 0.01, ns: not significant.

Next, we analyzed different histopathological and clinical characteristics for a potential influence on the patients’ overall survival: TPS, CPS, IRS, HPV status, primary tumor localization, and UICC stage. Thereby, PD-L1 expression of the primary tumor did not significantly affect overall survival in our cohort, whether assessed by CPS or TPS, as well as PD-L1-positive immune cells measured by IRS (Figure 5A–C). However, positive HPV tumor status was associated with significantly improved overall survival (Figure 5D; *p* = 0.0107). The subsite of the primary tumor also significantly affected overall survival, with tumors in the hypopharyngeal region showing the best overall survival (Figure 5E). In contrast with this finding, UICC stages did not significantly influence patient outcomes (Figure 5F). An additional analysis of disease-free survival yielded results that were largely consistent with those observed for overall survival (see Supplementary Figure S3). Multivariable survival analyses were performed using Cox proportional hazards regression in an exploratory framework, without formal adjustment for multiple comparisons, including age, sex, T stage, UICC stage, HPV status, PD-L1 expression (CPS ≥ 1), and immune infiltration (IRS low) as covariates. CPS ≥ 1 (HR = 34.74, *p* < 0.0001) and low IRS (HR = 9.017, *p* < 0.0001) were identified as independent predictors of worse disease-free survival, while positive HPV status (HR = 0.1831, *p* = 0.0007) was associated with significantly better DFS. For overall survival, no covariates were identified as significant predictors in multivariable analysis.

## 4. Discussion

Head and neck cancer is one of the most immune-inflamed human tumors, but response rates to PD-1 checkpoint inhibition, which represents the first-line treatment in recurrent and metastatic cases, are modest [21]. As shown in the IMCISION trial, there is not only an overall low response rate to anti-PD-1 treatment, but also a frequently varying probability of response between primary tumor and lymph node metastases [13]. We therefore analyzed PD-L1 expression in primary tumor samples and two matched lymph node metastases per case from 50 HNSCC patients treated at Saarland University Medical Center. By quantifying this inter-lesional divergence, we aimed to investigate whether such immunophenotypic heterogeneity potentially correlates with the mixed clinical responses observed across distinct anatomical compartments in the context of anti-PD-1 therapy. We observed clinical discordances in PD-L1 expression levels, using FDA and EMA approval cutoffs, between primary tumors and corresponding lymph node metastases, as well as among different lymph node metastases, in 18% (CPS; 95%-CI: 8.0–30.0%) and 4% (TPS; 95%-CI: 0.0–10.0%) of cases within our cohort. However, clinical variables such as HPV tumor status, T- and N-stage, and primary tumor localization did not significantly affect PD-L1 expression.

The heterogeneity of PD-L1 expression in head and neck cancer as a possible explanation for the poor response rate to immune checkpoint inhibition has been the focus of several studies over the past years. However, these studies primarily focused on the heterogeneity of intratumoral PD-L1 expression [11,22] or compared PD-L1 expression in a squamous cell cancer of unknown primary with oropharyngeal squamous cell carcinoma [23]. Comparable to our study design, Ambrosini-Spaltro et al., Schneider et al., and Deuss et al. have also investigated PD-L1 expression heterogeneity between primary tumors and lymph node metastases [24,25,26]. Ambrosini et al. reported similar findings on discordant PD-L1 expression between primary tumors and lymph node metastases, as measured by CPS, with 24% discordant cases, closely aligning with the 18% (95%-CI: 8.0–30.0%) observed in our study [26]. Conversely, Deuss et al. identified a higher discordance rate of 44% in their analysis. However, consistent with our findings, Deuss et al. observed no significant differences in CPS or TPS values between primary tumors and lymph node metastases [25]. The main differences between Ambrosini-Spaltro et al.’s work and ours lie in the number of patients included (Ambrosini-Spaltro et al.: n = 30, our study: n = 50) and the number of lymph node metastases analyzed (Ambrosini-Spaltro et al.: n = 17, our study: n = 100) [26]. In contrast to the study by Deuss et al., there were significant differences in the number of patients (Deuss et al.: n = 200, our study: n = 50) and the number of matched lymph node metastases (Deuss et al.: n = 33, our study: n = 50) [25]. Methodically, differences also occurred in the way of using tumor tissue samples. Deuss et al. employed tissue microarrays with 1.6 mm punches from the same HE slides to assess PD-L1 expression, while we used whole immunohistochemically stained slices, which is closer to the standard staining procedure in routine diagnostics and avoids potential sampling error. The unique feature of our study is the substantial number of matched lymph node metastases analyzed, which strengthens the validity of heterogeneous PD-L1 expression between primary tumors and lymph node metastases, as well as allowing for the comparison of PD-L1 expression in lymph node metastases with each other.

Regarding infiltration with PD-L1-positive immune cells, measured by the modified IRS, significantly higher score values were observed in lymph node metastases compared to the respective primary tumor. However, given the high density of immune cells within non-metastatic lymph nodes and the potential for intrinsic tissue-related bias, this finding is not unexpected and should be interpreted cautiously [27].

Although we observed no statistically significant differences in CPS and TPS values, tendencies towards varying scores between each respective site could be seen. Notably, varying CPS and TPS values were observed between the primary tumor and both lymph node metastases, as well as between the two lymph node metastases themselves. Similarly, Deuss et al. reported non-significant results with slight tendencies towards heterogeneous PD-L1 expression between primary tumor samples and lymph node metastases [25]. However, as mentioned before, methodological differences limit comparability to our study. Other HNSCC subgroup analyses by Kaur et al. revealed similar findings, showing discordant CPS values in 24% of cases between p16+ OPSCCs and one matched lymph node metastasis [28], which is close to our study, with 18% (95%-CI: 8.0–30.0%) of discordant cases. Brcic et al. reported a moderate correlation between TPS values of the primary tumor and lymph node metastases [29]. However, it is important to note that both studies evaluated either CPS or TPS to assess PD-L1 expression, comparing them to only one lymph node metastasis [28,29], but not both scores.

The clinical importance of our study is underlined by the percentage of concordant (CPS values consistently above or below 1 and TPS values consistently above or below 50% across all respective sites) and discordant cases of PD-L1 expression, respectively. Our findings show that 18% (95%-CI: 8.0–30.0%) of cases evaluated by CPS demonstrated site-dependent discordance, raising the possibility that eligibility for anti-PD-1 immunotherapy could vary according to the anatomical origin of the biopsy. These observations may indicate a clinically relevant opportunity to further explore whether targeted metachronous biopsy of lymph nodes could improve access to anti-PD-1 therapy, particularly in cases in which the primary tumor does not meet CPS ≥1 or TPS ≥50% thresholds. The clinical context of this consideration is exemplified by a real-world case from the Saarland University Medical Center. A 64-year-old patient, initially treated with curative-intent chemoradiotherapy for locally advanced HNSCC, presented one year later with suspected locoregional recurrence and lymph node metastases. Histology of the recurrent primary tumor showed CPS < 1, making the patient ineligible for immunotherapy under EMA guidelines. Due to contraindications for palliative chemotherapy, a targeted biopsy of a metastatic cervical lymph node was performed, revealing CPS > 10. Based on this result, pembrolizumab monotherapy was initiated and was well tolerated. This case suggests that PD-L1 heterogeneity may represent a potential explanatory factor for variable responses to immunotherapy and may, in certain circumstances, influence therapeutic eligibility and clinical decision-making when metastatic sites are re-biopsied. The observed discordance rate in our study is consistent with prior studies, which reported between 24% and 44% of cases exhibiting discordant PD-L1 status across sampling sites [25,26,28]. Potential explanations for the heterogeneous PD-L1 expression at different sites could be different compositions of the tumor microenvironment in the primary tumor and lymph node metastases [30] and potential changes in PD-L1 expression depending on disease stage [31], as well as the biological phenomenon of clonal evolution [32]. Notwithstanding these underlying mechanisms, the observed spatial heterogeneity in PD-L1 expression—both in our cohort and in prior studies—may have clinically relevant implications, potentially underscoring the importance of sampling site selection and sampling multiplicity in informing patient eligibility for immunotherapy. Importantly, the biological significance of PD-L1 dysregulation extends beyond oncologic contexts. Aberrant PD-L1 signaling has been implicated in the pathogenesis of inflammatory skin disorders, including psoriasis, atopic dermatitis, and cutaneous lupus erythematosus, where altered expression on keratinocytes and infiltrating immune cells contributes to the breakdown of peripheral tolerance [33]. Given the convergence of upstream regulatory pathways across malignant and immune-mediated conditions, mechanistic insights derived from HNSCC may inform therapeutic strategies in dermatologic disease, and conversely, advances in the latter may refine immunomodulatory approaches in cancer.

Regarding potential correlations of PD-L1 expression with clinical and pathological characteristics, HPV tumor status did not influence the CPS and TPS values of the primary tumor in our study. However, the role of HPV in PD-L1 expression is a topic of ongoing debate in the current literature [31,34,35]. Yang et al. and Chu et al. demonstrated a strong correlation between HPV positivity and increased tumoral PD-L1 expression [31,35], whereas Wang et al. found no significant correlation between PD-L1 expression and HPV tumor status, analyzing the TCGA-HNSC cohort [36]. Potential explanations for these partially contradictory results include various thresholds for defining positive cases [31], possible dynamic changes in PD-L1 expression depending on disease stage, and intratumoral heterogeneity in PD-L1 expression.

After accounting for tumor localization, we observed a trend towards higher CPS values in oropharyngeal cases compared with other subsites. These findings are consistent with those reported by Shestakova et al. in a similarly sized cohort (n = 57), although that study assessed PD-L1 expression using CPS alone [37]. Larger, site-balanced studies incorporating both CPS and TPS are warranted to further clarify potential localization-dependent differences in PD-L1 expression.

In terms of survival outcomes, PD-L1 expression had no correlation with overall survival in our study. Although PD-L1 expression has been established as a predictive biomarker for response rates to checkpoint inhibition therapy in numerous studies [38,39], the current literature remains unclear about whether PD-L1 expression is a prognostic biomarker outside of the context of immunotherapy. Our findings are consistent with many studies, including a meta-analysis by Yang et al. [40,41,42]. In contrast, other studies revealed improved overall survival upon high PD-L1 expression [25,43,44], while also negative associations between PD-L1 expression and patient survival have been reported [45,46]. Possible explanations for these conflicting findings include the use of different scoring systems (e.g., CPS, TPS) to define positive PD-L1 expression and the lack of standardized immunohistochemical detection methods, as well as heterogeneous patient cohorts.

The primary limitation of this study lies in the relatively small cohort size (50 patients), which precluded formal assessment of the proportional hazards assumption. Furthermore, the inherent heterogeneity of the patient population—specifically regarding tumor topography, with predominantly included oropharyngeal carcinomas, clinical staging, and gender-specific distribution—introduces potential confounding variables. While these data provide meaningful trends, the diversity of the cohort necessitates a cautious interpretation of clinical endpoints. Nevertheless, the inclusion of a total of 100 matched lymph node metastases did allow for the analyses of 3 matched samples per patient, making this the largest study of its kind in the current literature, to our knowledge. Prior studies have not analyzed this number of matched metastases of HNSCC cases, allowing for a detailed examination of PD-L1 heterogeneity between the primary tumor and lymph nodes, as well as among the lymph node metastases themselves. This extensive analysis provides valuable insights, although larger patient cohorts are still needed to further validate these findings and enhance clinical transferability. Secondly, country-specific regulations and guidelines for immune checkpoint therapy, particularly the CPS thresholds, present a significant challenge. Variability in regulatory approvals and clinical guidelines across different countries affects patient eligibility for immune checkpoint inhibitors. These discrepancies need to be considered when drawing conclusions from a result in clinical practice. Finally, it should be noted that our cohort consisted of patients receiving curative, surgical treatment, including only those patients with a resected primary tumor and at least two matching lymph node metastases. Owing to these stringent inclusion criteria, the distribution of tumor subsites in our cohort differs from that of some aforementioned immuno-oncology studies in head and neck cancer. Consequently, the extent to which findings derived from this cohort can be extrapolated to real-world populations of patients with recurrent or metastatic HNSCC, particularly with regard to therapeutic outcomes, may be limited.

## 5. Conclusions

Taken together, our findings suggest the presence of clinically relevant heterogeneity in PD-L1 expression between primary tumors and corresponding lymph node metastases, as well as among individual lymph node metastases themselves in head and neck cancer. These results may offer potential explanations for the varying immunotherapeutic responses of different lesions within the same patient, as observed in recent clinical trials [13,47,48]. This variability in PD-L1 expression suggests that future investigational strategies incorporating serial, multi-site biopsies alongside advanced spatial transcriptomic and single-cell profiling approaches will be required to delineate the mechanistic underpinnings of interlesional PD-L1 heterogeneity. Such efforts may further clarify whether dynamic re-sampling paradigms can meaningfully refine patient stratification and expand eligibility for immunotherapy. In this context, our study underlines that a deeper insight into and better understanding of the complex molecular background of PD-L1 expression is urgently needed to facilitate better prognostication and more effective as well as precise immuno-oncological treatment of head and neck cancer patients.

## Figures and Tables

**Table 1:** Clinical characteristics of included patients.

		HNSCC Patients
**No. of patients**		50
**Sex**	male	44 (88%)
	female	6 (12%)
**Median age (years)**		62.6
**HPV-Status**	positive	14 (28%)
	negative	36 (72%)
**Primary tumor**	oropharynx	30 (60%)
	larynx	9 (18%)
	hypopharynx	7 (14%)
	oral cavity	4 (8%)
**T stage**	1	5 (10%)
	2	23 (46%)
	3	11 (22%)
	4	11 (22%)
**N stage**	1	12 (24%)
	2	36 (72%)
	3	2 (4%)
**M stage**	0	46 (92%)
	1	4 (8%)
**UICC Stage**	I	7 (14%)
	II	6 (12%)
	III	0 (0%)
	IVa	33 (66%)
	IVb	1 (2%)
	IVc	3 (6%)
**Therapy**	surgery alone	3 (6%)
	surgery + RT	15 (30%)
	surgery + CRT	30 (60%)
	surgery + adjuvant RT + Cetuximab	2 (4%)

## Data Availability

The datasets generated during and/or analyzed during the current study are available from the corresponding author upon reasonable request.

## Supplementary Materials

The following supporting information can be downloaded at https://www.mdpi.com/article/10.3390/cancers18081286/s1: Figure S1: Prognostic impact of PD-L1 expression concordance; Figure S2: Subgroup analysis HPV; Figure S3: Disease-free survival; Table S1: Cox_OAS; Table S2: COX_DFS; Table S3: Mixed models.
