# Supplementary material for: Heterogeneity of PD-L1 Expression Between the Primary Tumor and Matched Lymph Node Metastases in Head and Neck Squamous Cell Carcinomas
Source: Cancers (Basel). 2026 Apr 18;18(8):1286. doi: 10.3390/cancers18081286 (PMC13114373; doi:10.3390/cancers18081286)
Supplement: Supplementary file 1 [file cancers-18-01286-s001.zip › cancers-4173603-supplementary.pdf]

cancers

Supplementary Materials

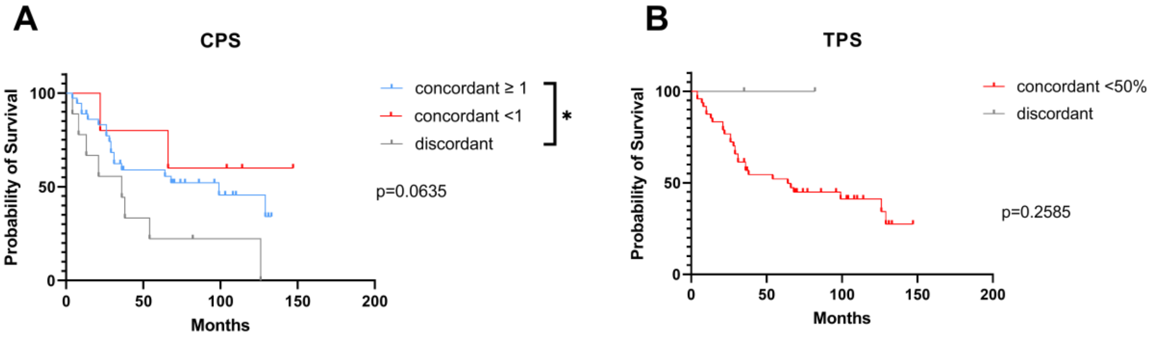

Figure S1. Prognostic impact of PD-L1 expression concordance between primary tumors and lymph node metastases (n=50) assessed by CPS (A) and TPS (B). Blue: CPS ≥ 1 or TPS ≥ 50% concordance. Red: CPS < 1 or TPS < 50% concordance. Grey: discordant expression between primary and metastatic sites.

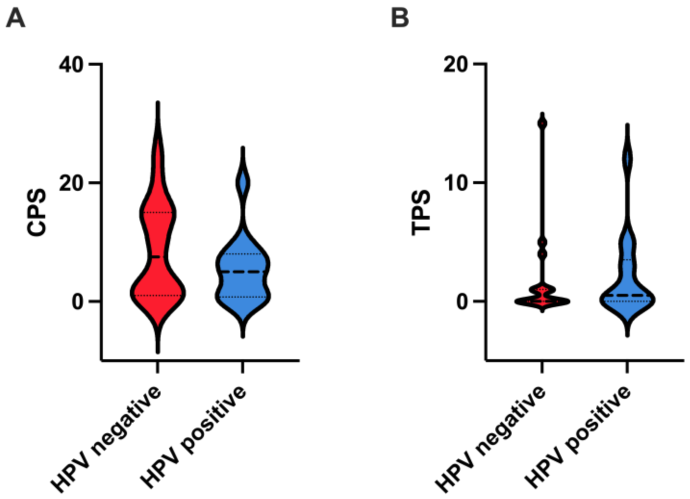

Figure S2. Subgroup analysis restricted to oropharyngeal carcinomas illustrating the association between HPV tumor status and PD-L1 expression in the primary tumor, assessed by (A) CPS and (B) TPS.

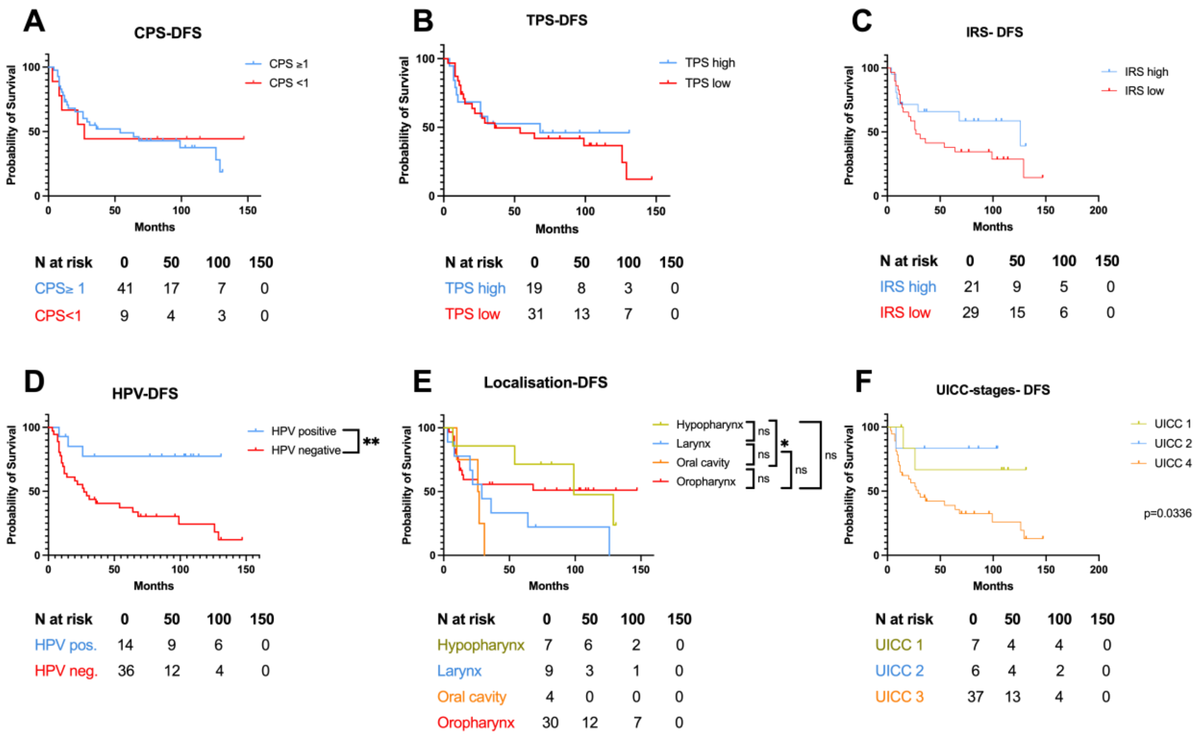

Figure S3. Disease free survival of CPS, TPS and peritumoral IRS of the primary tumor, HPV tumor status, primary tumor localization and UICC-stages. (A) Patients´ disease free survival regarding CPS values ≥ 1 and <1. (B+C) Correlation of disease-free survival with TPS values and peritumoral IRS values regarding high and low values, defined by the median. (D) Patients' disease-free survival segregated by HPV tumor status. (E) Disease free survival depending on localization of the respective primary tumor site. (F) Patient disease-free survival regarding different UICC stages. In (A) to (F), a log-rank test was used for statistical analysis. The number of patients at risk is provided in the corresponding table.

Table S1. Cox Proportional Hazards Regression Models for Overall Survival (OAS). Each model was adjusted for sex and age at diagnosis. Estimation method: exact. P-values from likelihood ratio tests. Event variable (OAS event) coded as 1 = death, 0 = censored. Note: all OAS models had zero censored observations.

Model	Variable	β (Est.)	HR	95% CI (HR)	LR Stat.	P value	n (Events)
1: CPS ≥1	CPS ≥1	−0.266	0.77	0.08–16.96	0.04	0.833	24 (24)
	Sex	−1.327	0.27	0.08–1.04	3.66	0.056	
	Age	0.006	1.01	0.95–1.06	0.04	0.836	
2: T stage	T stage 1	−0.201	0.82	0.13–2.95	0.07	0.787	29 (29)
	Sex	−0.955	0.38	0.13–1.41	2.21	0.137	
	Age	0.012	1.01	0.97–1.06	0.30	0.585	
3: HPV	HPV+	0.440	1.55	0.35–4.93	0.42	0.517	29 (29)
	Sex	−1.039	0.35	0.11–1.32	2.52	0.113	
	Age	0.014	1.01	0.97–1.06	0.38	0.540	
4: UICC	UICC	−0.107	0.90	0.25–2.61	0.03	0.854	29 (29)
	Sex	−1.001	0.37	0.12–1.40	2.28	0.131	
	Age	0.010	1.01	0.97–1.06	0.21	0.648	

5: IRS low	IRS low	0.454	1.57	0.22–31.96	0.17	0.683	22 (22)
	Sex	−1.227	0.29	0.07–1.43	2.44	0.118	
	Age	0.028	1.03	0.98–1.08	1.12	0.290	

No covariates reached statistical significance in any OAS model (all p > 0.05). Sex showed a consistent trend (p = 0.056–0.137) but did not reach the conventional threshold.

Model Diagnostics (AIC)

Model	AIC (empty)	AIC (model)	Δ AIC
1: CPS ≥1	99.1	101.3	+2.2
2: T stage	130.6	134.1	+3.5
3: HPV	130.6	133.8	+3.2
4: UICC	130.6	134.2	+3.6
5: IRS low	92.8	95.7	+2.9

All models show positive ΔAIC, indicating no improvement over the empty model, consistent with the absence of significant predictors.

Table S2. Cox Proportional Hazards Regression Models for Disease-Free Survival (DFS). Each model was adjusted for sex and age at diagnosis. Estimation method: exact. P-values from likelihood ratio tests. Event variable (DFS event) coded as 1 = recurrence/death, 0 = censored.

Model	Variable	β (Est.)	HR	95% CI (HR)	LR Stat.	P value	n (Events)
1: CPS ≥1	**CPS ≥1**	3.548	34.74	7.10–628.1	32.22	**<0.0001****	41 (26)
	Sex	−0.673	0.51	0.18–1.83	1.20	0.273	
	Age	0.017	1.02	0.97–1.06	0.51	0.476	
2: T stage	T stage 1	0.608	1.84	0.38–6.96	0.67	0.411	50 (31)
	T stage 2	0.505	1.66	0.64–4.76	1.04	0.309	
	T stage 3	0.111	1.12	0.33–3.72	0.03	0.855	
	Sex	−0.697	0.50	0.17–1.80	1.26	0.261	
	Age	0.004	1.01	0.96–1.05	0.04	0.851	
3: HPV	**HPV+**	−1.698	0.18	0.04–0.53	11.36	**0.0007*****	49 (31)
	Sex	−0.749	0.47	0.17–1.65	1.54	0.215	
	Age	0.004	1.00	0.96–1.05	0.05	0.831	
4: UICC	UICC	0.339	1.40	0.46–3.51	0.42	0.517	50 (31)
	Sex	−0.597	0.55	0.20–1.91	1.02	0.314	
	Age	−0.004	1.00	0.95–1.04	0.03	0.852	
5: IRS low	**IRS low**	2.199	9.02	2.90–39.91	17.07	**<0.0001****	32 (24)
	Sex	−0.719	0.49	0.14–2.23	0.99	0.319	
	Age	0.023	1.02	0.98–1.07	1.08	0.299	

**** p < 0.001; **** p < 0.0001. Proportional hazards assumption not formally tested (Schoenfeld residuals) due to small sample size.*

Model Diagnostics (AIC)

Model	AIC (empty)	AIC (model)	Δ AIC

1: CPS ≥1	152.4	124.2	−28.2
2: T stage	194.9	202.1	+7.2
3: HPV	193.5	186.7	−6.8
4: UICC	194.9	199.1	+4.2
5: IRS low	129.6	118.2	−11.4

Negative ΔAIC indicates improved model fit relative to the empty (no covariates) model.

Table S3. Linear Mixed-Effects Models (REML) for Repeated Measure. PD-L1 expression was compared across three matched specimen types per patient (PT = primary tumor, M1 = lymph node metastasis 1, M2 = lymph node metastasis 2; n = 50 patients). Sphericity was not assumed; Geisser–Greenhouse correction was applied. Post-hoc pairwise comparisons used Tukey's method.

A. Global Test (Fixed Effect: Specimen Type)

Outcome	F (DFn, DFd)	P value	GG ε	ICC	Matching P
TPS	F(1.90, 93.2) = 0.29	0.739 (ns)	0.951	0.35	<0.0001
CPS	F(1.63, 79.7) = 1.20	0.301 (ns)	0.814	0.54	<0.0001
IRS peritumoral	F(1.90, 93.1) = 8.71	**0.0004 (***)**	0.950	0.24	0.0048
IRS intratumoral	F(1.99, 97.4) = 13.54	**<0.0001 (****)**	0.994	0.20	0.018

GG ε = Geisser–Greenhouse epsilon; ICC = intraclass correlation (subject variance / total variance). Significant matching confirms within-patient correlation, justifying the repeated-measures design.

B. Post-Hoc Pairwise Comparisons (Tukey's HSD)

Outcome	Comparison	Mean Diff.	95% CI	Adj. P	Significance
TPS	PT vs. M1	−0.72	−3.26 to 1.82	0.774	ns
	PT vs. M2	−0.70	−3.08 to 1.68	0.758	ns
	M1 vs. M2	0.02	−2.85 to 2.89	0.999	ns
CPS	PT vs. M1	−1.82	−5.52 to 1.88	0.465	ns
	PT vs. M2	−0.04	−2.40 to 2.32	0.999	ns
	M1 vs. M2	1.78	−1.75 to 5.31	0.447	ns
IRS peri	PT vs. M1	−1.08	−1.83 to −0.33	**0.003**	**
	PT vs. M2	−1.21	−1.91 to −0.51	**0.0004**	***
	M1 vs. M2	−0.13	−0.98 to 0.72	0.927	ns
IRS intra	PT vs. M1	−1.48	−2.32 to −0.64	**0.0003**	***
	PT vs. M2	−1.61	−2.41 to −0.81	**<0.0001**	****
	M1 vs. M2	−0.13	−0.98 to 0.72	0.927	ns

*** p < 0.01; *** p < 0.001; **** p < 0.0001. IRS scores were significantly higher in lymph node metastases compared to primary tumors; no significant differences between M1 and M2.*

C. Random Effects and Model Fit

Outcome	SD (Subject)	Var (Subject)	SD (Residual)	Var (Residual)	REML Criterion

TPS	3.94	15.51	5.39	29.06	486.6
CPS	7.21	52.03	6.72	45.15	532.2
IRS peritumoral	0.90	0.82	1.59	2.53	300.5
IRS intratumoral	0.87	0.76	1.72	2.95	309.1
